# Modelling coupled human–environment complexity for the future of the biosphere: strengths, gaps and promising directions

**DOI:** 10.1098/rstb.2021.0382

**Published:** 2022-08-15

**Authors:** Isaiah Farahbakhsh, Chris T. Bauch, Madhur Anand

**Affiliations:** ^1^ School of Environmental Sciences, University of Guelph, Guelph, Canada; ^2^ Department of Applied Mathematics, University of Waterloo, Waterloo, Canada

**Keywords:** coupled human–environment systems, socio-ecological systems, regime shifts, social learning, social norms

## Abstract

Humans and the environment form a single complex system where humans not only influence ecosystems but also react to them. Despite this, there are far fewer coupled human–environment system (CHES) mathematical models than models of uncoupled ecosystems. We argue that these coupled models are essential to understand the impacts of social interventions and their potential to avoid catastrophic environmental events and support sustainable trajectories on multi-decadal timescales. A brief history of CHES modelling is presented, followed by a review spanning recent CHES models of systems including forests and land use, coral reefs and fishing and climate change mitigation. The ability of CHES modelling to capture dynamic two-way feedback confers advantages, such as the ability to represent ecosystem dynamics more realistically at longer timescales, and allowing insights that cannot be generated using ecological models. We discuss examples of such key insights from recent research. However, this strength brings with it challenges of model complexity and tractability, and the need for appropriate data to parameterize and validate CHES models. Finally, we suggest opportunities for CHES models to improve human–environment sustainability in future research spanning topics such as natural disturbances, social structure, social media data, model discovery and early warning signals.

This article is part of the theme issue ‘Ecological complexity and the biosphere: the next 30 years’.

## 1. The sixth extinction and the need for a CHES approach

**Figure 1.**
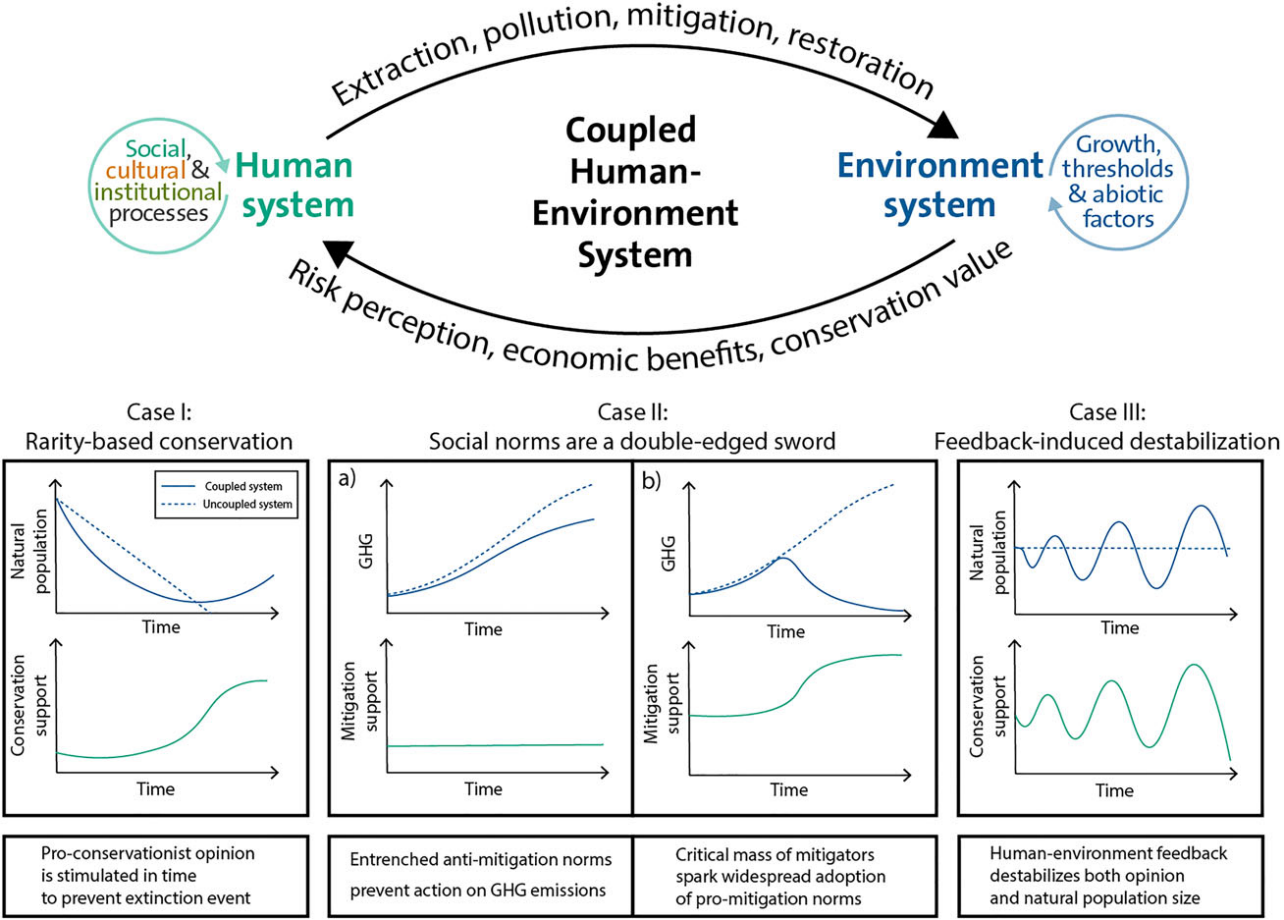
Case I: through rarity-based conservation, the human system responds to a declining natural population by increasing conservation support, reducing extraction which prevents collapse, and allows the natural system to recover; Case II: social norms which act to enforce majority behaviour can be both beneficial and detrimental to the health of the natural system, depending on the initial state of the social system; Case III: strong coupling between the human and natural system can lead to overshoot dynamics that destabilize an equilibrium with the potential to bring the natural system near extinction. (Online version in colour.)

Humans have a resounding impact on their natural environment, with anthropogenic disturbances being a leading factor in the Sixth Extinction. Ecological models usually represent human impacts on ecosystems through a fixed parameter representing a constant harvesting pressure or pollutant inflow, for instance. Under relatively short time scales, this can be a useful simplification, since human behaviour can be decoupled from the natural system and approximated as having a fixed rate of change. However, in any ecological system where coupling with a human system exists and the timescale of interest is sufficiently large, it may be necessary to abandon this assumption (figure 1). Instead, the framework of a coupled human-environment system (CHES) must be adopted, where the natural and human systems are coupled to form a single system. (Similar terminologies include socio-ecological systems, social-ecological systems and coupled human and natural systems). Human decision-making and behaviour play a crucial role in the dynamics of the natural system, while simultaneously being affected by changes in the natural system. As human and natural systems have become inextricably entwined, an approach that, at its core, acknowledges the two-way feedbacks present in these systems can help mitigate catastrophic events.

In the context of species extinctions, assuming fixed human behaviour on decadal timescales can lead to predicting more extinction events than have actually transpired. Human response can mitigate negative effects through efforts such as habitat conservation, pollution reduction and bioremediation. Pressure from the wider population, or groups of stakeholders, in response to dwindling natural species or ecosystems has often resulted in the preservation of that system and even a reversal of its downward course. An early example is pressure from Swiss citizens in the nineteenth century for cantons to halt deforestation, in response to flooding [1]. Subsequent examples include the rebound of the bald eagle population following the 1972 ban of DDT and introduction of conservation laws in the US sparked by scientific and public outcry [2,3], the recovery of wolf populations in Canada and the US following a shift in public perception and conservation laws [4], the development of the Northwest Forest Plan in response to changing public values calling for the preservation of old-growth forests [4,5], and the protection of large swaths of *Araucaria* forest in Southern Brazil by the government in response to extensive deforestation [6]. In other cases, small-scale harvesters have instituted social norms to prevent the worst effects of over-exploitation [7]. We refer to this response of populations at the nadir of a natural system as ‘rarity-based conservation’ (figure 1). This effect can be essential for understanding both the environmental and social conditions that lead to persistence or extinction of the natural system.

With specific reference to the Sixth Extinction, a 2050 time horizon suggests that modelling prospects and strategies for species and ecosystem conservation can benefit from capturing CHES interactions. Even when making accurate quantitative predictions over this timescale is difficult, such approaches can still be useful to compare different possible interventions, and evaluate how desirable they are relative to one another in terms of their qualitative benefits to sustainability. The possibility of gaining insights into how to produce sustainable outcomes in the presence of dynamic human–environment interactions is valuable.

## 2. The origins of CHES

Thomas Malthus presaged a role for environmental feedback when he proposed that human populations always grow exponentially until limited by (linearly growing) resource availability [8]. His work influenced Verhulst's logistic growth model, which describes exponential growth when resources are abundant and includes an environmental carrying capacity to represent the regime of resource-limited growth [9], first used to predict human population growth [10]. This same model was derived again a half-century later [11], soon being applied to predator-prey systems by Lotka & Volterra [12,13], whose work was seminal for ecology.

In the 1950s, motivated by the desire to maximize fishery yield while reducing the risk of collapse, bioeconomic models described single species fish populations undergoing logistic growth, where human harvest was represented by an effort parameter [14–16]. Subsequently, age structure with density-dependent mortality determined by harvesting effort was included [17]. By the 1960s, these models were developed into a dynamic framework where fish stock, harvest and effort varied through time as harvesters responded to changing profits in an open-access management framework, thereby becoming perhaps the first true CHES models [18]. Later fishery models included explicit spatial structure [19,20], increasing social complexity with the addition of individual agents that can follow iterative rules [21] and are even able to learn and base their decisions on limited available information using basic neural networks [22].

Another stream of early CHES models described the coupled dynamics of small, primarily indigenous human populations and local resources, inspired by Lotka–Volterra equations. It is important to note that many of these models were conceptualized from a white colonial perspective with many problematic assumptions and a lack of both consultation and consensual data acquisition. An early case study that was used in many of these models was the proposed self-regulating population dynamics of the Tsembaga Maring tribe in New Guinea through a ritual cycle that regulated their human population warfare, pig production and agricultural land use [23–25]. Interest in this vein of CHES modelling continued to grow with an influential model of the Rapa Nui population collapse [26–28]. A recent iteration of this model added the element of accumulated wealth while also partitioning the human population into elites and commoners, where elites prey on the wealth generated by commoners [29]. This model explores the ecological and socio-economic conditions leading to societal collapse in a CHES framework.

A third extensive category of CHES models studies the dynamics of land use, with a long history of using both social and ecological empirical data from landowners and tenants. The majority of these models account for spatial structure and localized interactions—something that is inherently important to land use and management. One of the earliest CHES land use models defined interactions between plots of land and mobile tenants through land use, carbon release and settlement diffusion dynamics [30,31]. Others parametrize land transitions using location and environmental characteristics [32]. These early models focused primarily on the environmental conditions leading to land transitions, but subsequent studies adopted a CHES approach using multi-agent models, where independent actors, sometimes parametrized by socioeconomic data, make decisions regarding the state of their parcel of land with limited information and social learning [33–36]. Additional heterogeneity was introduced in the types of agents' interactions, with both landowners and institutional actors [37]. These models have increased in complexity, for example by including water flows, crop and vegetation dynamics coupled to social management practices that govern water, land, capital and a labour force [38].

Common-pool resources are defined as being open access and finite, such as some forests and fisheries, and other examples in the preceding paragraphs. Elinor Ostrom played a foundational role in framing, studying and modelling these systems within a CHES framework through her research around how human populations self-organize and allow for the maintenance and persistence of common-pool resources in the absence of a central governing body. One of her main findings was the importance of social norms, which are shared understandings of acceptable behaviour. Through both theoretical and empirical studies, Ostrom posited that these norms can lead to long-lasting cooperative behaviour, especially if enforced through sanctions [7,39–41]. An early example incorporating social norms into a CHES model is for a human population harvesting a common-pool resource [42]. Here, the dynamics of resource users, denoted by their strategies as cooperators (mitigators), defectors (non-mitigators) and enforcers (who sanction defectors) are dependent on both the state of the social and environmental system, modelled using techniques from evolutionary game theory.

## 3. How are social processes modelled?

As social processes in CHES models may involve strategic decision-making of individuals, many models drawing inspiration from game theory, formalized in [43]. Since its initial focus on one-shot games with two players, the field has developed in many ways, such as exploring opinion dynamics in populations. These dynamical models stem from evolutionary game theory, which combines the classical framework with biological models of evolution, and thereby confers a temporal dimension to individual interactions and decision-making. Rather than focusing on the strategy a rational player should choose, there is greater emphasis on how the frequency of strategies in a given population changes throughout time.

**Figure 2.**
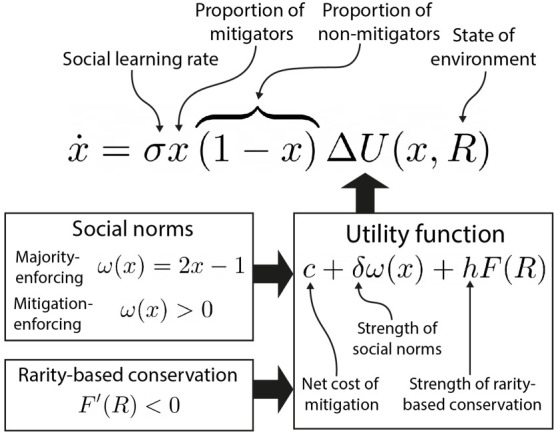
Replicator dynamics is a common theoretical framework for modelling the human system. In CHES, the replicator equation (top) usually represents the rate of change of the proportion of mitigators, *x*. The relative utility of mitigation is determined by the utility function, ΔU (right), which often includes terms representing the net cost of mitigation, *c*, social norms, ω, and rarity-based conservation, *F*. The speed of social dynamics relative to the environment is represented by σ, which can equivalently be controlled through a similar term in the environmental system.

Models that describe the aggregate population dynamics often represent human dynamics using replicator equations [44] (figure 2). Here, each individual samples other's traits at a fixed rate, changing their trait only if it appears to offer a higher utility than their current trait. The utility function may include a parameter for the net cost of mitigation, for instance, which acts as an incentive or deterrent for adopting the mitigator ‘trait’ (opinion), depending on its sign. Social norms can also be included. Norms that simply enforce the majority behaviour can act as a double-edged sword, with the ability to incentivize both mitigative and non-mitigative behaviour, depending on the currently dominant norm. Whereas, mitigation-enforcing norms only confer benefit to mitigators or equivalently incur sanctions to non-mitigators, which increase with the current frequency of mitigators. Utility terms for rarity-based conservation cause the utility to adopt a mitigator opinion to increase as the environment approaches collapse, unlike the fixed net cost of mitigation. Finally, the rate of social learning determines the speed at which social change occurs, relative to the environment dynamics.

Stochastic decision-making is also used to represent social dynamics and is often applied in the context of best response dynamics, where players choose the highest-utility strategy for the current state of the system (instead of relying upon social learning). In a stochastic framework, probabilities of changing strategies are represented with logistic functions that include a term for the difference in utility between strategies as well as a parameter that tunes the degree of stochasticity [45,46]. These are used in both agent-based models and ODE models of population dynamics [47]. A third approach to social dynamics is threshold models, where agents choose to participate in an action based on their individual threshold for the number of people already participating [48,49]. These models allow for a focus on the social structure of a given population, as two groups with the same mean threshold to participate could have drastically different dynamics given the distribution of thresholds among individuals. Threshold models have also been formulated for continuous systems where the frequency of participants is modelled through population dynamics [50,51] and have been sparsely applied to CHES models [52,53]. For agent-based models, there are a number of ways in which individuals learn, often inspired by voter models [54] or Ising models [55] where agents simply imitate the majority opinion of their peers. These can increase in complexity to include utilities associated with replicator equations, but we will not discuss these in detail since our focus is on the replicator equation approach. For a review of agent-based learning models, see [56,57].

## 4. Insights, strengths and weakness of CHES

In the following sections, we review findings among relevant contemporary studies in the CHES literature. These studies were found from a keyword search on Google Scholar using ‘human environment system’ OR ‘socio-ecological system’ OR ‘social ecological system’ OR ‘human ecological system’ OR ‘human natural system’ combined with ‘coupled model’ OR ‘dynamics’ OR ‘theory’ OR ‘social learning’ OR ‘social norms’ OR ‘conservation’ OR ‘time horizon’ OR ‘time discounting’ OR ‘foresight’. Additional literature was found through works cited by relevant papers.

### (a) Systems in isolation versus CHES approach

To demonstrate the profound impact of human feedbacks in CHES, many studies have directly compared CHES models to the uncoupled environmental system, where dynamic human influence is replaced with fixed parameters. In all cases, CHES feedback leads to a richer number of possible regimes—and regime shifts—that are absent in the uncoupled model [58–63]. Often, this coupling can stabilize the environment, allowing resources to persist for longer than expected under the constant harvesting assumption [60,61,64]. CHES feedback can also alter the relationship between environmental variables and cause counter-productive outcomes. For example, in a decoupled climate model, a low solar flux will lead to lower peak temperatures, however with human-climate feedbacks, this slower temperature increase will incentivize humans to become non-mitigators, who in turn emit enough additional greenhouse gases that peak temperatures increase to higher levels than seen under baseline solar flux levels [65].

### (b) Human response to the environment

Increasing the ability for humans to respond to the threat of environmental collapse through effects like rarity-based conservation often leads to beneficial outcomes, as seen in a coral reef model [66], forest-pest models [67,68] and generalized resource models [69,70]. For lake eutrophication models, increasing the strength of rarity-based conservation was found to reduce pollution levels [47,71]. A more recent model demonstrated a stronger effect of rarity-based conservation, where increasing levels led to higher mitigation, with the ability to destabilize a high pollution equilibrium through stable limit cycles. At very high levels of rarity-based conservation, a stable low pollution equilibrium emerged [72]. In many models, however, increasing the strength of human coupling can have counterintuitive outcomes. For example, in forest models, strong rarity-based conservation effects can destabilize an equilibrium of full forest cover, giving rise to oscillatory dynamics in both opinion and forest cover [60,73]. This results from humans valuing conservation only when the environment is near depletion, leading to scenarios where humans harvest at high levels until the forest is near depletion and lower their extraction rate until forest cover rebounds (figure 1, Case III). These dynamics increase the risk of collapse in the environment system. Rarity-based conservation has also been modelled as one of many environment-dependent social incentives, in which increasing its level led to mixed strategy and pure mitigation equilibria, bistability and stable limit cycles depending on the strength of the other incentives [74].

Along similar lines, in a fishery model where the strength of resource dependence on harvesting efforts was varied, increasing coupling strength led to an increase in the dominant period of the limit cycles, followed by chaotic dynamics. Intermediate coupling levels were also associated with a higher resource yield, declining as coupling was further increased [61]. Additionally, coupling has been represented by an individual's perception of the environment, where if the environment passes below a threshold of degradation, individuals will become alarmed and reduce their extractive effort [75], similar to rarity-based conservation. Here, lower levels of coupling led to a higher biomass equilibrium, offering long-term environmental benefits, whereas with high levels of coupling, the system passed through a higher minimum state of biomass, offering short-term benefits. Similarly, in a fishery-pollution model, health concern functions as a form of coupling as it informs the demand for pollution abatement. Among the model's findings were that decreasing the level of health concern can lead to a pollution epidemic so long as the fishing industry is able to persist [76].

### (c) Enviromental response to humans

CHES models usually represent the strength of coupling of the environment to the human system through the harvest rate. In many models, including those for forests, generalized resources and fisheries, high harvest rates can lead to oscillations in both the state of the environment and human opinion [59,61,73,74]. It can also have contradictory effects, where in a forest cover model it could lead to collapse or benefit the environment, depending on other social parameters [60]. Through an alternative modelling approach, with nonlinear coupling and a constantly increasing efficiency of harvesting to represent technological growth, increased coupling led to faster resource collapse at lower levels of harvesting efficiency [77]. Similarly, in an Earth system model where humans could harvest from two energy sources, increased coupling to biomass through harvest led to a collapse of the environmental system [78]. One study examined the strength of coupling through reducing the strength of strictly ecological terms in the environmental system. For three generalized resources, increasing the strength of coupling benefited sustainable outcomes, but also obscured differences between the natural systems, with all three models displaying oscillatory dynamics in both the environment and human system for high levels of social learning [62].

### (d) Social learning

In input-limited models, such as human-managed resource extraction, high social learning rates tend to destabilize equilibria. Some examples are land use [62,79,80], coral reef [66] and generalized resource models [74,81,82], where faster learning leads to oscillations in both the human and environment system. An earlier agent-based land use model found different outcomes through an alternative approach to learning, where high information flow between agents led to a lower average harvesting rate and higher levels of forest cover [83]. Similar work in this vein shows that higher social learning rates lead to instability, causing synchronized harvest among landowners with rapidly declining forest cover followed by gradual recovery [84,85]. On the other hand, slower rates of social learning have been shown to benefit sustainable outcomes, increasing the stability of the high forest cover [53], and supporting mitigators for a generalized resource [86]. In adaptive network models, where each node represents a renewable resource with an associated harvest effort, low rates of social learning and increased homophily were most effective in leading to a sustainable equilibrium, and a transition between sustainable and unsustainable equilibria occurred when the rate of social learning was approximately equal to the rate of ecological change for homogeneous resources [87,88].

In output-limited models, where human behaviour contributes to a detrimental environmental process such as forest-pest and climate systems, high social learning rates lead to better mitigation of environmental harms in the short term [65,67,68,89], however these high learning rates are not always sufficient for long-term sustainable outcomes without additional interventions and often have diminishing returns as they are further increased. Contrasting this, in lake eutrophication models, high social learning rates destabilized equilibria and led to limit cycles [47,72,90]. A recent extension to these models found this to be the case only with strong social and no ecological hysteresis, however high social learning could also stabilize oscillations in conditions of no social and strong ecological hysteresis [71]. Alternatively, social learning can occur through imitating similar agents' decision-making (how information is used to decide a strategy) rather than the strategy itself. One land use model found that adding this feedback, paired with longer term decision-making, resulted in a significant change in the type of agriculture developed, leading to higher household wealth [91].

In some cases, the strength of social learning has system-dependent contradictory effects. For example, a global land use model showed that increasing the strength of social learning increased agricultural land use under low incentives for an eco-conscious diet and low future yields. However, if incentives favoured adopting an eco-conscious diet and future yields are high, increased social learning decreased land use [92]. In a model of deforestation through ranching, higher rates of social learning which led to faster intensification of ranching only resulted in higher deforestation for a stable cattle market, with a long-term reduction in deforestation resulting under a saturating market [93]. Only one recent CHES model showed invariance under varying rates of social learning [69] and this has been attributed to the fact that the resource in this system does not contain intrinsic dynamics and instead has a human-dependent growth term [74].

### (e) Social norms

In CHES models, strong majority-enforcing social norms typically lead to extreme equilibria consisting of a single strategy, determined by the initial frequency of strategies. This double-edged effect with the potential to support both sustainable and catastrophic outcomes has been found in forest-pest, forest cover, coral reef and climate change models (figure 1, Case II) [60,65–68]. Increasing the strength of these norms has also been shown to increase the number of regimes and generate alternative stable states [80].

Increasing the strength of mitigation-enforcing social norms often benefits sustainable outcomes [94–96]. However, in a generalized resource model, very high levels of these norms led the population to harvest at suboptimal levels [96]. This was also shown in a fishery model, however for low levels of social norms, slight over-harvesting by mitigators could make the system immune to invasion by defectors leading to higher long-term sustainability [97]. In many lake eutrophication models, strong social norms consistently led to high levels of mitigation and low levels of pollution, having a greater impact when the system was already in a state of high mitigation [47,71,90]. A similar model found increasing these norms led to the appearance of alternate stable states, while decreasing the likelihood of collapse [72]. In a generalized resource system, decreasing the strength of social norms caused a catastrophic regime shift to resource overexploitation, where re-establishing a population of mitigators was very difficult or infeasible [58]. In a forest cover model, the direction rather than the strength of the social norms was varied through a global parameter, where increasing this norm toward overharvesting led to a decrease in the amount of robust forest cover [83]. Social norms as sanctions can also be resource-dependent, becoming more severe when the environmental system is close to collapse, combining both the effects of social norms and rarity-based conservation [98].

### (f) Net cost of mitigation

Decreasing the net mitigation cost often has a beneficial effect on the environment, by increasing the proportion of mitigators and moving the system away from less stable oscillations, as seen in forest cover and coral reef models [60,63,80,99]. However, in a forest cover model, initial conditions with low mitigator frequency led to oscillations in opinion and forest cover before the system reached a stable state, as the cost of mitigation was increased [80]. A contradictory effect was found in a lake eutrophication model where decreasing the cost of mitigation led to high mitigation and low pollution levels with a higher positive impact for initial conditions of low mitigation [47]. On the other hand, increasing mitigation cost can lead to a catastrophic regime shift with very low levels of mitigation and hysteresis, making it difficult to restore the system to its previous state [58]. In recent human–climate models, the cost of mitigation can be changed simultaneously with other social parameters such as the social learning rate, to accelerate mitigation [65,100]. In a common-pool resource model, agents have their payoff reduced relative to their harvest effort by a cost per unit effort parameter, which acts similarly to a negative mitigation cost. Here, high levels lead to the persistence of the resource even when individuals are motivated by profit over sustainability goals [101].

### (g) Foresight

Many CHES models account for foresight in the human decision-making process. In models of forest cover, pollution, ecological public goods and reinforcement learning, this environmental foresight can be very significant in conserving natural states or mitigating harmful action [52,65,73,81,83,102]. One forest-grassland model included an additional term for economic foresight, finding the persistence of the forest-grassland mosaic to be highly dependent on individuals valuing long-term environmental health over long-term economic benefits [73]. In many cases, the foresight of social groups can change with time and in response to the state of the environment, as explored through a climate change model where each country's foresight in policymaking was treated as a dynamic social trait influenced through imitation [103].

### (h) Strengths and weaknesses of CHES: a summary

In summary, we have discussed a brief history of CHES modelling, comparing insights across studies, and touched on several strengths and weaknesses of this framework (table 1). Perhaps the most fundamental strength of CHES modelling is its ability to represent dynamics in systems where human and environment respond to one another, which is an increasingly prevalent situation during the Sixth Extinction. These two-way feedbacks at the core of CHES models have been observed in many empirical case studies (see §1) and when modelling similar systems, classical ecological models that lack these feedbacks will have more limited application, especially on sufficiently long time horizons. Including CHES feedbacks often leads to richer model behaviour, allowing for valuable insight into both sustainable and catastrophic trajectories, and a comparison of the possible interventions and how human societies will respond to them. The diversity of interventions primarily stems from the ability to represent human behaviour mechanistically at many levels (e.g. social norms, rarity-based conservation), which can in turn elucidate the process of human behaviour and choice. Finally, CHES models benefit from generality, with the ability to apply similar models of human behaviour to disparate human-environment systems. In some cases, these techniques have been applied to entirely different fields, such as epidemiological modelling [104,105].

**Table 1.** A comparison of the CHES and classical ecological modelling frameworks through their strengths and weaknesses.

	classical ecological models	CHES models
strengths	easier to create a detailed representation of environmental processes	provides mechanistic representation of human-environment feedbacks that dominate many systems
	simpler dynamics	rich dynamical regimes
	more limited data requirements	provides valuable insight into the effect of human interventions
	easier model validation and analysis	
weaknesses	human role can be oversimplified the point of unrealism for many systems	requires data from both human and environment systems
	does not provide insight into how human interventions respond to environmental changes	higher dimensionality, thus more difficult to analyze
		requires data on coupling terms, which does not always exist

Some weaknesses of CHES modelling include model complexity challenges stemming from representing both human and environment systems mechanistically. Addressing this ‘curse of dimensionality’ by opting for a simplified CHES model can result in a lack of heterogeneity in both the social and ecological systems. On the other hand, retaining complexity of both human and environment representations can make model analysis difficult, and requires more data than just an environment model or just a human model. Additionally, social data are lacking in some systems that would permit parameterization and validation, which reduces the predictive power of these models (although the coming years will likely see an improvement in this situation). Many of these weaknesses will be further addressed in the subsequent section, with suggestions for improvement. For example, future CHES models could represent more relevant psychological and social processes, using social media data to permit model parameterization.

## 5. Gaps and promising future directions in CHES modelling

### (a) What can we learn from ‘uncoupled’ developments on each ‘side’?

We can advance CHES modelling not only by improving our understanding of the coupling, but also by harnessing recent progress in ecology and using more sophisticated representations of human systems. Here we outline some of the major developments on both of these sides.

Disturbances contribute significantly to ecosystem dynamics, and natural disturbances can either be an essential aspect of ecosystem structure and function, or have devastating effects. There is a large knowledge gap regarding interacting disturbances and their ability to have unpredictable and catastrophic effects. With the increase of anthropogenic disturbances, improving our understanding is essential for mitigating the worst effects of the Sixth Extinction and finding sustainable trajectories into the future. Disturbance interactions have been categorized as ‘linked’—altering the likelihood, extent, or intensity of subsequent disturbances—or ‘compound’—altering the recovery time or trajectory of an ecosystem with the potential to create novel disturbances that can drastically affect ecosystem resilience [106]. A novel framework combines discontinuous shocks to the system with continuous dynamics to allow for modellers to explore repeated disturbances across a wide variety of systems. It also offers new methodology to measure resilience to these disturbances, proposing metrics based in disturbance space that reflect the dynamic interplay between disturbance and recovery [107]. Under this framework, linked disturbance interactions would increase the frequency and intensity of shocks, whereas compounded disturbance interactions would alter the trajectory of the flows. This can be applied to human systems as well, where shocks are discrete perturbations in the frequency of opinions caused by events or opinion leaders, and flows could be the dynamics of the replicator equation. In CHES, the direction of flows in human opinion between shocks in either subsystem could influence the persistence of the environment as a flow towards non-mitigation could compound with harmful shocks, bringing the system to a state of non-mitigator dominance and/or resource collapse. On the other hand, a flow towards mitigation between shocks could reduce their harmful effects, improving the resilience of the resource. Additionally, shocks in the human system could respond to the environment, representing immediate responses of rarity-based conservation, triggered by natural disasters and/or the enactment of new environmental legislation.

Many CHES models view human populations as relatively uniform in how they learn and interact. However, demographic structure can play a significant role in how individuals learn and respond to their environment, as well as influencing human population size. Some recent CHES models have introduced demographic structure, particularly through the partitioning of human populations by economic class, for example in coupled climate models [89,100,108] and a human population model [29]. Other recent examples are the partitioning of a human population into resource users who only change their harvesting strategy in response to the environment, and users who are also susceptible to social influence [75], as well as a model with multiple social functional traits relating to agricultural management capacities, which demonstrated that social diversity enhanced ecosystem services [109].

Age structure is much less explored in the CHES literature, although it is known that age can significantly affect environmental concern, action and the mechanisms of learning [110–113]. Climate change action, for instance, is clearly a generational phenomenon [111]. These diverse responses to environmental issues can profoundly influence CHES dynamics and can be modelled through age-dependent learning, mitigation rates, information sources and responses to policy. Theoretical models agree with empirical observations of imitation at a young age followed by individual learning [114,115], and more recently, models have shown that fast environmental changes select for individual-based learning [116], whereas parental learning is optimal in slowly changing environments [117,118]. Age has also been modelled as a trait that determines social influence [119–121], with a recent study showing that varying the influential age group affected the frequency of cooperation [122].

Biases can also play an important role in human learning. For example, confirmation biases [123] can be represented through the bounded confidence opinion model [124,125], which represents individual opinions as continuous values, only allowing agents to update their opinions with information that is within a given threshold away from their own opinion [126]. Another learning bias that can be modelled is conviction [127,128], which can be seen as opposite to the rate of social learning, and has been used as an individual trait in a land use CHES that used a modified bounded confidence model [129]. These biases can be associated with the demographic structure of a population, as seen in [130,131], which modified the voter model to include age-dependent conviction.

The environmental and social impact of institutions, as well as their rates of change, are strikingly different than those of most individuals, yet there remains largely unexplored potential to account for these differences in CHES models (agent-based models especially). One recent global model addressed this by exploring policy shifts between local and global agreements for climate change mitigation [132], finding a well-timed shift from local to global agreements could show significant benefit in mitigating climate change, compared to other approaches. Additionally, most CHES models assume a discrete set of strategy choices that humans can adopt, however in reality these opinions evolve over continuous spectra. The bounded confidence [124,125] and DeGroot models [133] are two well-known approaches that account for a continuous strategy space and have been used to model online social networks [134,135], including a mass media environment [136,137].

### (b) Incorporating new data

In earlier CHES models, social data for model parameterization was generated in collaboration with social scientists through population surveys [138–140]. Since the rise of online social networks and mass media, there is now a plethora of observational data for online human interactions, as well as the emergence of social models that aim to represent novel dynamics that arise in these environments.

Some models have drawn from the bounded confidence and DeGroot models and incorporated homophily on networks (echo chambers), [141], to fit trends in online data, while classifying users by personality types [142–145] and differentiating sources of opinion change [146]. Sentiment analysis of social media datasets, used to parametrize and validate some of these models, has been broadly applied in topics from voter preference for the 2008 US election [147], to beliefs on vaccination and climate change [148,149]. This can be extremely helpful to CHES modelling, as public perception of extreme environmental events can be quantitatively analysed [149], and metrics quantifying the likelihood of transitioning between social media states can detect anomalies in public perception [150]. Social media data has also been used to reconstruct user networks based on interactions and examine homophily within these networks [148], which can inform CHES network models. Empirical trade and transport networks can provide important insights into the spatial CHES coupling as well as human metapopulation models [151], having already been applied to CHES models for invasive species [152,153] and land use via global food trade [92,154].

For the ecological side of CHES models, a trait-based approach could adapt existing generalized models to specific case studies, ideally with environmental data, to improve their effectiveness for scientists and policymakers. Recent advances using techniques such as machine learning [155], transfer functions [156], and trait-dependent carrying capacities [157] show great promise incorporating plant trait data into models that use parameters that are difficult to measure such as recruitment, growth and interaction strength. Plant trait models have already proven to be very effective in accounting for realistic ecological dynamics [158] and these traits can be useful in the prediction of novel interactions (e.g. invasive species, biological control) through data that is readily available [159]. Along with existing plant-trait databases (e.g. [160,161]), other ecological datasets can be immediately applied to CHES models such as an invasive plant dataset with associated bioclimatic variables [162], a database of ecosystem services [163] as well as land use datasets that already contain human environment coupling that can further motivate future models [164–166].

### (c) Model discovery

Often when constructing mathematical models for real world processes, specific details such as precise functional forms between variables and equation parameters cannot be accurately known. This is an important limitation in CHES modelling, which requires knowledge of both the feedbacks within and between two distinct complex systems. In such cases, generalized modelling can give insight into many crucial aspects of the system while leaving many details unspecified [167,168]. Although this technique lacks the ability to generate predictive time series, it can identify the types of regime shifts that are possible, and assess the stability of equilibria [58,64]. Additionally, these generalized models can be related to empirical systems through generalized modelling parameters that require less data than traditional approaches and can help elucidate ambiguity in underlying mechanisms [64]. This approach has shown promise when used for a coupled resource harvesting model [58] and a fisheries model [64,169]. Similarly, machine learning has been applied to predict regime shifts from time-series data, without specification of the complex feedbacks that cause them, while also predicting the type of bifurcation to expect [170]. This technique could be used in tandem with generalized modelling to analyze the stability and potential trajectories of very complex, highly-dimensional CHES for which we have time-series data but lack mechanistic understanding. Another recent advance uses sparse regression and a library of candidate nonlinear functions to find a system of differential equations that best describe time series data with the fewest terms [171,172]. To our best knowledge, this technique has yet to be applied to CHES models, however a similar model discovery approach was implemented for a land use change and water resource use model [173].

### (d) Early warning signals

Regime shifts in natural systems can be catastrophic, especially in the presence of hysteresis, which can bring the system into a depleted state from which it is extremely difficult to recover. Many systems display particular types of early warning signals when approaching a regime shift [60,174–178]. Near tipping points, these systems demonstrate a decreased resilience to disturbances, taking a longer time to return to their stable equilibrium if perturbed. The added complexity brought about through coupling human behaviour to environmental systems leads to a greater number of regime shifts in CHES systems, which makes interpreting early warning signals much more challenging, especially in predicting the new regime after a transition. Furthermore, the addition of the social system can mute commonly used early warning signals, making these transitions even more difficult to predict [60]. Offsetting this drawback, many CHES models have found signals from the social system to be much more effective in predicting regime shifts than similar metrics gathered from the environmental system [58,60,77,170]. This shows great potential as both social media and economic systems generate immense amounts of real-time data, which could be used to monitor environmental systems and improve our understanding of regime shifts through incorporating this data into CHES models. Similarly, socio-ecological network models have demonstrated that properties of the social network can affect the accuracy of early warning signals [179,180], further motivating the need to better understand the structure and role of human systems and their adaptive feedback, especially with regard to mitigating the many looming environmental catastrophes that humanity currently faces.

## 6. Concluding comments

We have reviewed the history and recent developments of CHES models, demonstrating their importance to understanding the diverse and sometimes surprising outcomes of complex interactions between humans and the environment. The CHES approach reveals novel regimes and trajectories that we would not know from environmental models alone. In many cases, these feedbacks can reveal new paths to more sustainable outcomes as humans respond to the threat of environmental collapse in the Sixth Extinction. However, feedbacks that are too strong can have destabilizing effects, leading to oscillatory dynamics in both subsystems. Additionally, the effects of interventions in CHES can lead to drastically different outcomes depending on the type of environmental system. The effect of social norms, if majority-enforcing, are very sensitive to the initial makeup of the social system and can act as a double-edged sword, encouraging the status quo, regardless of the implications for sustainability. Foresight in decision-making, however, has shown to be beneficial across a vast array of systems and model formulations. Insights from this more holistic modelling approach can inform policymakers as to which interventions will be most effective in mitigating potential catastrophic trajectories and ushering in a more sustainable future. This is perhaps best seen through the power of social interventions, as CHES models have demonstrated that their relationship to the environment and timing is very important.

Given the immense environmental impact of social action, it is useful to evaluate the extent of social change necessary to mitigate environmental disasters such as climate change and loss of biodiversity. Although incorporating human feedback into models helps in the understanding of CHES, it is still difficult to predict exactly what will happen. It is clear that in scenarios when the human system accelerates a harm such as pollution or the spread of an invasive species, the rates of social change are too slow. For example, we have known about greenhouse gas emissions leading to climate change for decades, but unfortunately, the global response has been insufficient to mitigate its worst effects [104,105]. By coupling climate models to a human system, we were able to show that in order to meet IPCC goals [181], social learning must be fast enough for our entire population to change their current behaviour within five years of the model's publication, which seems infeasible. For realistic sustainable trajectories, social change needs to be supported through well-timed institutional change such as policies that reduce the cost of mitigation as well as improved foresight [65]. Plotting sustainable trajectories in other vulnerable CHES is extremely urgent and requires further research. Regarding the global challenges faced by declining biodiversity, there are currently no models for the social action needed to reach biodiversity goals set out by the IPBES [182]—a gap in our knowledge that requires immediate study. Ultimately, there are limits to what can be known through modelling. However, qualitative insights are extremely useful to inform policymaking that can successfully mitigate future environmental catastrophes. Implementing model findings poses a great challenge that future CHES modellers will need to address.

## Data Availability

This article has no additional data.
